# RNAi silencing of c-Myc inhibits cell migration, invasion, and proliferation in HepG_2_ human hepatocellular carcinoma cell line: c-Myc silencing in hepatocellular carcinoma cell

**DOI:** 10.1186/1475-2867-13-23

**Published:** 2013-03-08

**Authors:** Yan Zhao, Wang Jian, Wei Gao, Ya-Xin Zheng, Yong-Kun Wang, Zhu-Qing Zhou, Hui Zhang, Cong-Jun Wang

**Affiliations:** 1 Department of Gastroenterology, Shanghai Tenth People Hospital, Tongji University School of Medicine, Shanghai, 200072, PR. China; 2 Department of General Surgery, Shanghai East Hospital, Tongji University School of Medicine, Shanghai, 200120, PR. China

**Keywords:** C-Myc, Hepatic cancer, HepG_2_ cells, RNAi, siRNA

## Abstract

**Background:**

Hepatocellular carcinoma (HCC) is the most common type of liver cancer. Although much is known about both the cellular changes that lead to HCC and the etiological agents responsible for the majority of HCC cases, the molecule pathogenesis of HCC is still not well understood. We aimed to determine the effect of c-Myc gene expression on the proliferative, invasive, and migrative capabilities of hepatocellular carcinoma HepG_2_ cells.

**Methods:**

A plasmid- based polymerase III promoter system was used to deliver and express short interfering RNA targeting c-Myc to reduce its expression in HepG_2_ cells. Western blot analysis was used to measure the protein level of c-Myc in HepG_2_ cells. The effects of c-Myc silencing on the invasion, motility, and proliferation of HepG_2_ cells were assessed using a Transwell chamber cell migration assay system and a growth curve assay, respectively.

**Results:**

The data showed that plasmids expressing siRNA against c-Myc significantly decreased its expression in HepG_2_ cells by up to 85%. Importantly, pSilencer-c-Myc transfected cells showed a significantly reduced potential in migration, invasion, and proliferation.

**Conclusion:**

C-Myc plays an important role in the development of hepatocellular carcinoma. The data show that down-regulating the c-Myc protein level in HepG_2_ cells by RNAi could significantly inhibit migration, invasion and proliferation of HepG_2_ cells. Thus, c-Myc might be a potential therapeutic target for hepatocellular carcinoma.

## Background

Hepatocellular carcinoma (HCC) is the most common type of liver cancer. Most cases of HCC are secondary to either a viral hepatitis infection (hepatitis B or C) or cirrhosis (alcoholism being the most common cause of hepatic cirrhosis) [1]. Although much is known about both the cellular changes that lead to HCC and the etiological agents responsible for the majority of HCC cases (hepatitis B virus, hepatitis C virus, alcohol), the molecule pathogenesis of HCC is still not well understood [2,3].

Myc (c-Myc) is a regulator gene that encodes for a transcription factor. A mutated version of Myc is found in many cancers, which causes Myc to be constitutively (persistently) expressed. This further induces an unregulated expression of several genes, some of which are involved in cell proliferation [4]. A frequent genetic abnormality seen in HCC is the overexpression of c-Myc [5,6]. The importance of c-Myc expression in HCC is demonstrated both by studies of transgenic mice [7,8] and clinical research which has indicated that overexpression of c-Myc is found in most HCC patients and correlated with poor prognosis [9].

A recent study demonstrates that temporary inhibition of Myc selectively kills mouse lung cancer cells or induces apoptosis, making it a potential cancer drug target [10,11]. In the human genome, Myc is located on chromosome 8 and is believed to regulate expression of 15% of all genes [12] through binding on Enhancer Box sequences (E-boxes) and recruiting histone acetyltransferases (HATs). This means that in addition to its role as a classical transcription factor, Myc also functions to regulate global chromatin structure by regulating histone acetylation both in gene-rich regions and at sites far from any known genes [13]. In this study, we aimed to investigate whether specific down-regulating the protein level of c-Myc in a HepG_2_ cell line might result in the inhibition of cell growth.

RNA interference (RNAi) is a process in which activation of an intracellular pathway modulated by small-interfering RNA (siRNA) composed of 21–23 nucleotides (nt) leads to degradation of a specific, targeted mRNA [14]. The selective and robust effect of RNAi on gene expression makes it a valuable research tool, both in cell culture and in living organisms because synthetic dsRNA introduced into cells can induce suppression of specific genes of interest [15,16]. RNAi has been used to inhibit diseases induced by virus (for instance, HIV [17] and influenza [18]), tumorigenesis caused by oncogenic K-ras, and H-ras [19,20], activation of oncogenes resulting from chromosomal translocations (for example bcr/abl in chromic myeloid leukemia [21]), cancers caused by viral infections, and others. Recently, retroviral-based approaches to deliver siRNA into tissue cultured mammalian cells have been proved to be powerful, and doxycycline-regulated inducible gene expression by RNAi has been shown to be particularly useful for the analysis of genes that are necessary for cellular survival (22,23). These studies have established a new area in the genetic manipulation of human cancer development by allowing oncogenes to be down regulated by RNAi. For this purpose, RNAi directed against c-Myc was used here.

In this study, HepG_2_ cell was transfected with a plasmid which was under the control of the U6 promoter to deliver and express siRNA targeting c-Myc to determine whether this technique could be used for the specific inhibition of oncogene overexpression and whether this inhibition resulted in antitumor effects. Our results showed that specific down-regulation of c-Myc by RNAi was sufficient to inhibit the proliferative, invasive, and migrative capabilities of HepG_2_ cell, and that c-Myc might serve as a therapeutic target for HCC.

## Results

### Selection of siRNA transfectants

HepG2 cells were co-transfected with pSILENCER 1.0-U6 generating short hairpin siRNA and with pEF6/V5-His TOPO expression vector carrying selectable blasticidin marker as described in the Materials and Methods section. Transfected cells were selected for blasticidin resistance and characterized by the presence of the insert in the genomic DNA (data not shown).

### Suppression of c-Myc expression in HepG2 cells by RNAi

**Figure 1:**
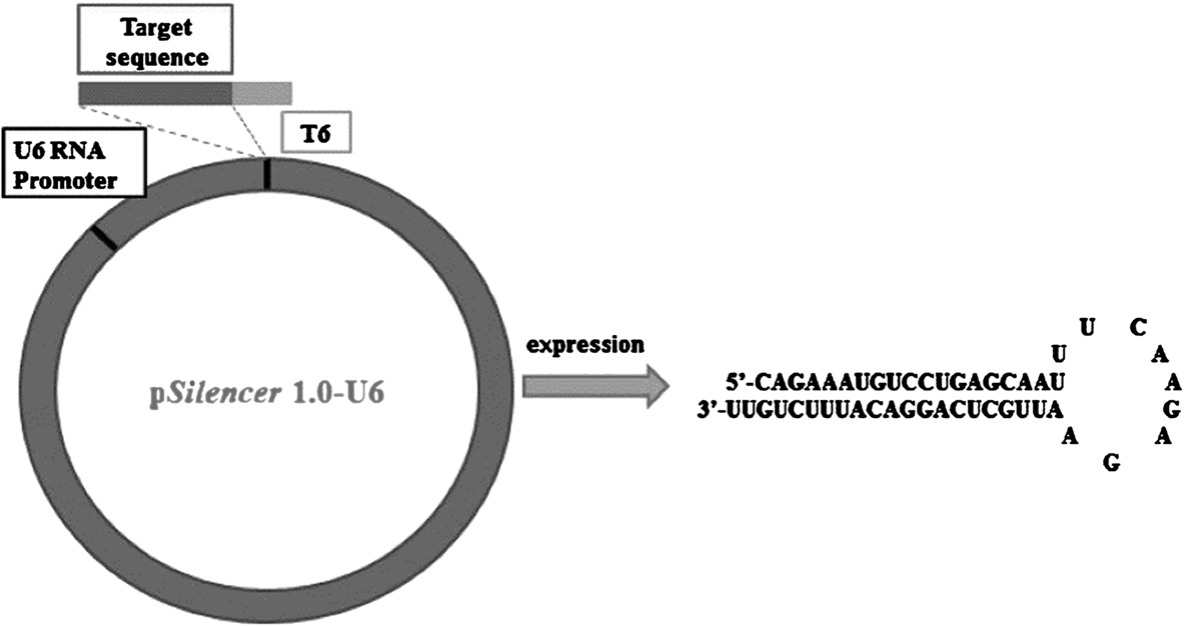
**Schematic drawing of the pSliencer 1.0-U6 vector.** The U6-RNA promoter was cloned in front of the gene-specific targeting sequence (19-nucleotide sequences from the target transcript separated by a short spacer from the reverse complement of the same sequence) and six thymidines (T6) as a termination signal.

One pair of oligonucleotides encoding short hairpin trans cripts directed against a portion of the c-Myc mRNA was designed and synthesized. This pair of oligonucleotide was then ligated into pSilencer. The plasmid of pSilencer–c-Myc contains a U6 promoter that directs the synthesis of oligonucleotides in an inverted repeat with 9 nt for its loop, with six T bases added at the end to serve as a termination signal for RNA polymerase III. The RNA is expected to fold back to form a hairpin loop structure after being transcribed; the hairpin dsRNA can then be further cleaved by Dicer to generate a 21-nucleotide siRNA, the active form for the RNAi effect, which will form dsRNA–endonuclease complexes and will bind and destroy c-myc mRNA inside cells [17] (Figure 1).

**Figure 2:**
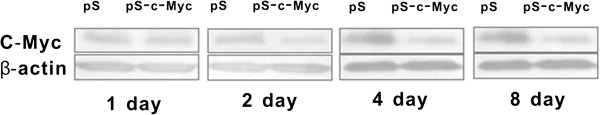
**Time course of the reduction in c-Myc protein levels by pSilencer-c-Myc (pS-c-Myc).** Exponentially proliferating HepG_2_ cells were transfected with pS–c-Myc or p*Silencer (pS)* and whole cell lysates were prepared at the time points indicated. Total cell lysates were separated by SDS–PAGE and immune blotted with an antibody against c-Myc; expression levels were normalized for loading by probing for β-actin.

PSilencer–c-Myc was transfected into HepG2 cells and its effects on c-Myc protein levels were determined by comparison with pSilencer-transfected cells by western blot at the time points indicated. We found that the c-Myc expression levels were suppressed by up to 85% in HepG_2_ cells at day 4 after transfection. Actually the levels of c-Myc were decreased as early as 24 hours after transfection and remained at low levels until 8 days after transfection (Figure 2). Our obtained inhibition rate by siRNA seemed to be higher than that in previous report (67%), which demonstrated better cell transfection efficiency in our study [11]. The inhibitory effect was shown to be specific because transfection with pSilencer did not alter c-Myc levels. In addition, RNAi did not cause a nonspecific down-regulation of gene expression, as determined by the β-actin control (Figure 2). These data indicated that vector-based RNAi could effectively suppress c-Myc overexpression and resulted in prolonged decreases in specific cellular gene expression without marked effects on other cellular proteins.

### Effect of siRNA c-Myc on cell invasion, motility, and proliferation

**Figure 3:**
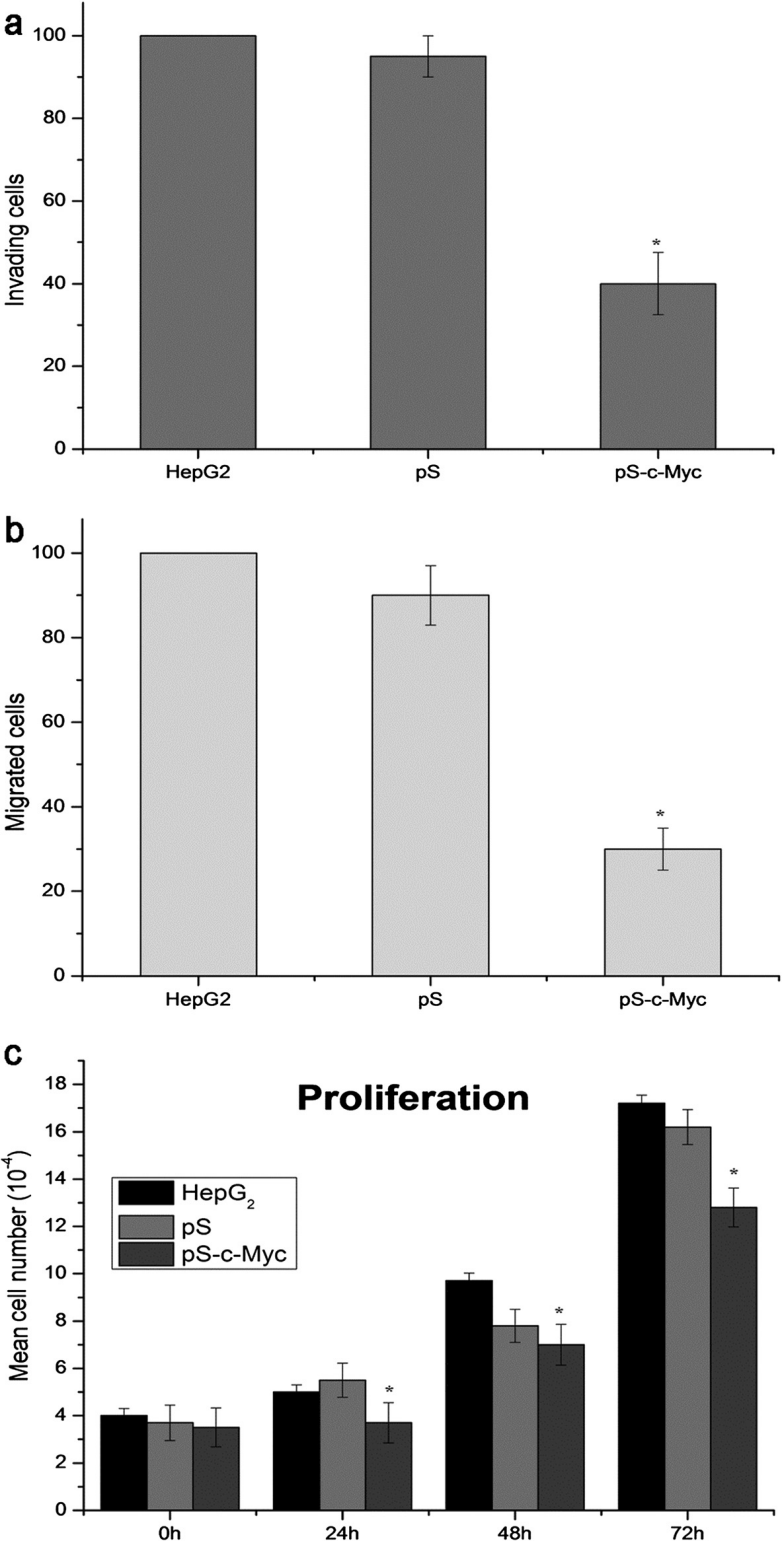
**Invasion (a), motility (b) and proliferation (c) of parental, vector, and siRNAi-vector transfected cells.** The percentages of invading (t=24 hours) and migrating (t=24 hours) cells were calculated as reported in Materials and Methods. The pictures highlighted the differences in number between HepG2, pS, and pS-c-Myc cells able to invade (a) or migrate (b) to the lower surface of transwells. RNAi directed against c-Myc led to a reduced cellular growth rate after 24, 48 and 72 hours. Columns, averages of three independent experiments; error bars, SD. *P <0.05.

The *in vitro* invasion assay was designed to test whether transfection of siRNA c-Myc altered the invasive potential of tumor cells in Matrigel-coated transwell chamber. Considering as 100% in the untreated HepG2 cells which are able to invade to the filters in 24 hours, the invasive percentage of vector alone–transfected cells was 83% and that of siRNA c-Myc–transfected cells was 27% (Figure 3a). The invasive potential of pS siRNA c-Myc cells significantly decreased (P < 0.05).

To study the effect of siRNA c-Myc transfection on migration, parental and transfected cells were seeded on transwell chambers with uncoated filters. After 24 hours of incubation, the motility potential of pS siRNA c-Myc cells was significantly reduced (Figure 3b; P < 0.05).

To determine changes in the growth pattern of HepG_2_ cells stably transfected with siRNA c-Myc, a growth curve was done on them along with parental cells and the cells transfected with the empty vector. c-Myc inhibition by siRNA c-Myc transfection modulated the proliferation of hepatocellular carcinoma cells; in fact, the proliferation of pS siRNA c-Myc cells was markedly decreased compared with parental and vector-alone–transfected cells (P < 0.05) (Figure 3c), indicating that c-Myc is involved in the proliferative potential of hepatocarcinoma cells.

In summary, inhibition of endogenous c-Myc using siRNA technology could reduce the motility, invasion, and proliferation of HCC cells. Although we obtained comparable c-Myc inhibition with plasmid expressing antisense c-Myc mRNA, the advantages provided by siRNA technology should be relevant for in vivo studies avoiding nonspecific defense responses against duplex RNA longer than 30 nt [18-20]. In addition, future studies should be performed to test the effect of c-Myc silencing on the other HCC cells.

## Conclusions

Our findings confirm that down-regulation of c-Myc may be an effective way of achieving a significant reduction in the malignant properties of HCC-derived cells and imply the therapeutic potential of RNAi on the treatment of hepatocellular carcinoma by targeting overexpression oncogenes such as c-Myc.

## Materials and methods

### Reagents

RPMI 1640 medium (Life Technologies, Inc., Gaithersburg, MD); pEF6/V5-HisvTOPO vector (Invitrogen; the plasmid containing the blasticidin-selectable marker gene); DOTAP Liposomal transfection reagent according to the manufacturer’s instructions (Invitrogen); anti-c-Myc (9E10; 1:1000 dilution; Santa Cruz) and anti-β-actin (AC-15; 1:5000 dilution; Sigma), anti-mouse IgG–horseradish peroxidase conjugate antibody (Zhongshan Company); Luminol chemiluminescence detection kit (Santa Cruz).

### Cell line and cell culture

The HepG_2_ human cell line was provided by the Shanghai Cell Research Institution of Chinese Academy of Sciences. The cells were cultured with RPMI 1640 medium supplemented with 100 mL/L fetal calf serum, 1*10^5^ U/L penicillin and streptomycin at 37°C in a 5% CO_2_ incubator.

### Design and preparation of constructs

To design siRNA targeting c-Myc, one annealed set of oligonucleotides (for 3 minutes at 90°C and for 1 hour at 37°C) encoding short hairpin transcripts corresponding to 1906–1926 nt of c-Myc mRNA (GenBank accession no. NM-002467) [21] were cloned into pSilencer 1.0-U6 (containing ampicillin resistance gene and the human U6 RNA Polymerase III promoter). The short-hairpin-RNA-encoding complementary single-stranded oligonucleotides, which hybridized to give overhangs compatible with ApaI and EcoRI, were designed with a computer program available on the Internet (http://www.ambion.com/techlib/misc/psilencerconverter.html.) Oligonucleotides encoding short hairpin RNAs were then ligated into pSilencer. Bacterial colonies were pooled and used for plasmid preparation. The positive clones were confirmed by sequencing. The resulting plasmid was designated as pSilencer–c-Myc. The sequence of the insert was confirmed by automated sequencing and analyzing the fragments generated from digestion with *Hind*III.

### Transfection of cells

HepG_2_ cells were grown to 60% to 80% confluency (in 6- cm-diameter Petri dishes) and then co-transfected with 10 ug of pSILENCER 1.0-U6 and 1 ug of pEF6/V5-HisvTOPO vector using DOTAP Liposomal transfection reagent. Transfected cells were selected for blasticidin resistance (6 ug/mL; pS: cells transfected with vector-alone; pS siRNA C-Myc: cells transfected with siRNA against C-Myc).

### Western blot analysis

The 24-hour serum-free conditioned media (2 mL/6-cm diameter Petri dish) were collected from confluent cultures of parental, pS, and pS siRNA c-Myc transfected cells. Constant amounts of proteins from conditioned media of transfected and non-transfected cells were loaded in SDS-PAGE, under non-reducing conditions, on a polyacrylamide gel composed of three layers, containing different acrylamide concentrations. Gel was blotted on nitrocellulose membranes and immune reacted with antibodies. The antibodies and dilutions used were anti-c-Myc (9E10; 1:1000 dilution; Santa Cruz) and anti-β-actin (AC-15; 1:5000 dilution; Sigma). After being washed extensively, the membranes were incubated with anti-mouse IgG–horseradish peroxidase conjugate antibody (Zhongshan Company) for 1 hour at room temperature and developed with Luminol chemiluminescence detection kit (Santa Cruz). Membranes probed for β-actin was used to normalize for loading and/or quantification errors and to allow comparisons of target protein expression. Protein expression was quantified with a Gel EDAS analysis system (Cold Spring USA Corporation) and Gel-Pro Analyzer 3.1 software (Media Cybernetics).

### Invasion and motility

Invasion assay was done in a 24-well transwell chamber. Cells were added to coated filters in 100 μL of serum-free medium in triplicate wells. In the lower compartments of the chambers, 600 μL of human fibroblast-serum-free-conditioned media was used as chemo attractant. After 24 hours at 37°C in a 5% CO_2_ incubator, the Matrigel coating on the upper surface of the filter was wiped off using a cotton swab. Cells that migrated through the filters were fixed, stained with Hema-3, photographed, and counted. The motility assay was conducted in a similar procedure. Cells were loaded on transwell polycarbonate membrane inserts in triplicate wells. The plates were incubated for 24 hours at 37°C in a 5% CO_2_ incubator, the cells in the lower wells were fixed, stained with Hema-3, and counted. The cells that had migrated to the lower compartment of the chambers were trypsinized and counted. Each experiment was carried out in triplicate. The statistical significance of the results was calculated using the ANOVA procedure. The data were considered to be significant when P < 0.05.

### Proliferation assay

HepG_2_ and HepG_2_-transfected cells were seeded in triplicate in 3-cm-diameter Petri dishes. After 24, 48, and 72 hours of incubation at 37°C, the cells were trypsinized and counted using a Burker chamber.

## Abbreviations

HCC: Hepatocellular carcinoma; RNAi: RNA interference; HATs: Histone acetyltransferases

## Competing interests

This project is supported by Grants from the National Natural Science Foundation of China (No.30872510, 81200320, 81272534, 81260349), the Natural Science Foundation of Hubei Province (No.2008CDB127), and the Natural Science Foundation of Shanghai (No.064119620 10411968400). This work is funded by the Health System Key Discipline Group of Pudong New District (PWZXKQ2010-5), and funded by the Foundation of Shanghai Tenth People Hospital (12XSGG105). There are no any conflicts of interests.

## Authors’ contributions

Yan Zhao and Jian Wang participated in the design of this study, and they both performed the statistical analysis. Wei Gao carried out the study, together with Ya-Xin Zheng, Yong-Kun Wang collected important background information, and drafted the manuscript. Zhu-Qing Zhou, Cong-Jun Wang and Hui Zhang conceived of this study, and participated in the design and helped to draft the manuscript. All authors read and approved the final manuscript.
